# Sonographic findings using the SAFE-A protocol in pre- and post-hemodialysis patients

**DOI:** 10.1186/s13089-024-00390-5

**Published:** 2024-08-27

**Authors:** Matheus Rabahi, Maria Goretti Polito, Larissa Louise Cândida Pereira Takaoka, Marcus Barreto Conte, Philippe Figueiredo Braga Colares

**Affiliations:** 1 Departamento de Medicina Interna, Hospital Estadual Alberto Rassi, Goiânia, GO Brazil; 2 Departamento de Nefrologia, Hospital Estadual Alberto Rassi, Goiânia, GO Brazil; 3 Departamento de Medicina Interna, Hospital de Emergência de Goiânia, Goiânia, GO Brazil; 4 Unidade de Pesquisa Clínica, Faculdade de Medicina de Petrópolis/UNIFASE, Petrópolis, RJ Brazil; 5 Departamento de Pneumologia, Instituto do Coração, Hospital das Clínicas, Escola de Medicina, Universidade de São Paulo, São Paulo, SP Brazil

**Keywords:** Ultrasonography, Intravascular volume status, Hemodialysis

## Abstract

**Background:**

Accurate assessment of relative intravascular volume is one of the cornerstones for the proper management of hospitalized patients requiring hemodialysis. Currently, the use of dynamic parameters such as bedside ultrasonography is recommended to support the assessment of the intravascular volume profile. This study aimed to prospectively evaluate findings of sonographic assessment of intravascular volume estimate (SAFE-A) protocol among hemodialysis inpatients with end-stage renal disease, before and after the hemodialysis sessions, and correlate these findings with the net ultrafiltrate (UFNET).

**Results:**

A positive correlation was found between the negative variation of 1 point in the score of the SAFE-A protocol with the withdrawal of 426.73 mL of net ultrafiltrate.

**Conclusions:**

There was a strong correlation between the score of the SAFE-A protocol and the net ultrafiltrate. Therefore, this study concludes that the application of the SAFE-A protocol in dialysis patients demonstrates a correlation between the suggested score and volume status, consistent with findings from the original study conducted in a distinct population.

## Background

Accurate assessment of relative intravascular volume is a cornerstone for the proper management of hospitalized patients who require hemodialysis due to acute kidney injury (AKI) associated or not with end-stage renal disease (ESRD). These patients frequently have intradialytic hypotension (IDH) with symptoms of target-organ ischemia and a need for clinical interventions. Furthermore, IDH may limit volume removal by ineffective ultrafiltration, prolonging hemodialysis time and/or increasing the number of sessions [1].

To improve these patients’ hemodynamic management different methods have been described to assess the relative intravascular volume such as the inferior vena cava (IVC) ultrasonography [2, 3] and the “5B” approach, i.e. balance of fluids (reflected by body weight), blood pressure, biomarkers, bioimpedance vector analysis, and blood volume [4].

To better evaluate the intravascular volume profile, the use of dynamic parameters such as volume correction by inferior vena cava collapsibility index (IVCCI) is currently recommended [5, 6], since it reflects the volemic status in patients with congestive heart failure [5] or undergoing hemodialysis [7]. Additionally, the rate of pulmonary B-lines disappearance as the volume is removed is used to better assess the dry weight of hemodialysis patients [8–10]. Moreover, echocardiography has been considered important to identify the causes of circulatory instability in the presence of numerous hemodynamic conditions [11]. Finally, the assessment of the internal jugular vein collapsibility index (IJVCI) gives an excellent overview of the circulatory collapse [12–15]. Joining these four variables, the sonographic assessment of intravascular volume estimate (SAFE) was developed to estimate intravascular fluids classifying patients according to their intravascular volemic status [11]. SAFE protocol includes ultrasonographic assessment of cardiac contractility, inferior vena cava and internal jugular collapsibility indices, and pulmonary B-lines. Also, pulmonary B-lines assess extravascular volume, while cardiac contractility does not evaluate relative intravascular volume.

This study aimed to prospectively evaluate findings of SAFE-a protocol among hemodialysis inpatients, before and after the hemodialysis sessions, and correlate these findings with the net ultrafiltrate (UFNET).

It is worth mentioning from the outset that the original study, the hypovolemic, normovolemic, and hypervolemic profiles were scored as -1, 0, and + 1, respectively. In the present study, we adapted them to + 1, +2, and + 3. Therefore, it was termed the Adapted SAFE Protocol (SAFE-a).

## Methods

### Patient selection

Inpatients with 18 years or older undergoing hemodialysis three times a week for at least three months, fully performing the hemodialysis session, and spontaneously breathing were invited to take part of the study. Were excluded inpatients with a clinical suspicion or a confirmed diagnosis of severe heart disease, systemic sclerosis, interstitial lung disease of any nature, acute pulmonary infection, ongoing neoplasm at any site, deformities of the rib cage preventing ultrasound evaluation, a pulmonary, hepatic, or heart transplant. Thirty patients were recruited for the study.

### Data collection

After signing a written informed consent and immediately before the beginning of the hemodialysis session, we collected sociodemographic data, history of comorbidities, and recent laboratory tests. Subsequently, an ultrasound examination was performed by an experienced bedside ultrasound operator (MR), trained and certified by the World Interactive Network Focused On Critical UltraSound, who operates the equipment on a daily basis, following the SAFE protocol (Killu et al., 2020) [11], and using a portable ultrasound device model Butterfly iQ+ (Butterfly Network, Inc., Guilford, CT, United States). The images were stored and reviewed by two physicians, a pulmonologist and a nephrologist, also experienced in bedside ultrasound.

For each organ, the modes and frequencies in the handheld ultrasound device were altered as follows: for the cardiac examination, the cardiac mode was used, with a frequency ranging from 5.0 to 7.5 MHz, depending on the patient’s adipose tissue; for the pulmonary examination, the linear transducer mode was employed; for IVC, the curvilinear transducer mode was used; for the examination and measurements of IJV, the headboard was raised at a 30-degree angle and the linear transducer mode was employed, placed laterally at the level of the cricoid cartilage. We used the following formula to calculate IVCCI and IJVCI:

$$\:\left[\frac{\left(\text{m}\text{a}\text{x}\text{i}\text{m}\text{u}\text{m}\:\text{d}\text{i}\text{a}\text{m}\text{e}\text{t}\text{e}\text{r}-\:\text{m}\text{i}\text{n}\text{i}\text{m}\text{u}\text{m}\:\text{d}\text{i}\text{a}\text{m}\text{e}\text{t}\text{e}\text{r}\right)}{\text{m}\text{a}\text{x}\text{i}\text{m}\text{u}\text{m}\:\text{d}\text{i}\text{a}\text{m}\text{e}\text{t}\text{e}\text{r}}\right]\times\:\:100$$

The same sonographic protocol was applied immediately after the hemodialysis session. In addition, we collected some data after the hemodialysis session such as ultrafiltrate (UF), UFNET, medium blood pressure, and adverse events.

On this study, the following scores were assigned: cardiac function – (a) hyperkinetic = 1; (b) normal = 2; (c) hypokinetic = 3; pulmonary evaluation – (a) < 1 B-lines mean per field = 1; (b) 1–2 B-lines mean per field = 2; (c) ≥ 3 B-lines mean per field = 3; IVC diameter and spontaneous respiratory variation – (a) < 2.5 cm in widest diameter and > 50% respiratory variation in diameter = 1; (b) 1.5–2.5 cm in widest diameter and < 50% respiratory variation in diameter = 2; (c) > 2.5 cm in widest diameter and < 50% respiratory variation in diameter = 3; IJV – (a) > 40% respiratory variation = 1; (b) 20–40% respiratory variation = 2; (c) < 20% respiratory variation = 3. The scores of all four exams were added up to have a final score (SAFE score) for compound of cardiac contractility, extravascular pulmonary edema, and relative intravascular volume, and finally interpreted as: (a) 4 to 6 = hypovolemia; (b) 7 to 9 = normovolemia; (c) 10 to 12 = hypervolemia.

Regarding the cardiac evaluation methodology of the SAFE protocol, it was strictly followed by the researcher, utilizing the same steps that included obtaining a long-axis view of the heart, observing cardiac function, and estimating ejection fraction using either the eyeballing method or M-mode with maximum systole and diastole measurements; additionally, and also a short-axis view of the heart was obtained to assess cardiac function and estimate ejection fraction.

It is important to mention that in the SAFE protocol, the number of B-lines counted from all examined segments was added together, and then divided by the total number of segments examined to calculate the average. In our study, the same approach was employed, with scanning performed in 4 lung regions for each hemithorax.

In the original study, the hypovolemic, normovolemic, and hypervolemic profiles were scored as -1, 0, and + 1, respectively. In the present study, we adapted them to + 1, +2, and + 3. Therefore, it was termed the Adapted SAFE Protocol (SAFE-a).

### Data analysis method

We characterized the demographic profile, hemodialysis session data, and sonographic findings using absolute frequency and relative frequency for categorical variables and mean and standard deviation for continuous variables. We verified data parametricity using the normalized Q-Q plot and standardized residue histogram [16].

We evaluated the distribution of ultrasound findings before and after hemodialysis by applying the McNemar test followed by post-hoc analysis [17]. We compared the SAFE-A score before and after hemodialysis using the paired t-test and performed multiple regression analysis between the UFNET with the variation of the score of the SAFE-A protocol (ΔSAFE-A), variation of the number of B-lines (Δnumber of B-lines), variation of the echocardiography (Δechocardiography), variation of the inferior vena cava (ΔIVC), and variation of the internal jugular vein (ΔIJV). We adopted the Backward method to select the model with greater accuracy and predictive power and the Pearson’s correlation matrix to evaluate the relationship between the variations. We analyzed data applying the Statistical Package for Social Science version 26.0 (IBM SPSS Statistics for Windows, IBM Corporation, Armonk, NY, United States) and the significance level of 5% (*p* < 0.05)

### Ethical

Informed written consent was obtained from all participants and the study was apprioved by the Institutional Review Board (IRB) of the Hospital Estadual Alberto Rassi (Goiânia, GO, Brazil) on August 18, 2022 (CAAE: 59768822.3.0000.0035).

## Results

From August 20 to December 15, 2022, 46 subjects were screened and 30 were enrolled on this study. 16 subjects were excluded because due to incomplete hemodialysis sessions Table 1 shows social and demographic data and some clinical characteristics of the participants. Table 2 shows the parameters assessed during the hemodialysis sessions, while Table 3 displays all the parameters of the SAFE-A protocol before and after the hemodialysis session.

**Table 1:** Social and demographic profile and some clinical characteristics of the 30 participants, in Hospital Estadual Alberto Rassi, Goiânia, GO, Brazil, from August to December 2022

Baseline characteristic	*n*	%
**Sex**		
Female	13	43.3
Male	17	56.7
**Age group**		
21 to 59 years	18	60.0
60 to 82 years	12	40.0
**Already received kidney transplant**		
No	22	73.3
Yes	8	26.7
**Indication for hemodialysis**		
ESRD, started hemodialysis in the last 3 months	17	56.7
ESRD, on hemodialysis for more than 3 months	13	43.3
**NYHA functional classification ≥ 3**		
No	29	96.7
Yes	1	3.3
**Arteriovenous fistula**		
Right upper limb	2	6.7
Left upper limb	2	6.7
No	26	86.7

*n* absolute frequency, *%* relative frequency, *ESRD* end-stage renal disease, *NYHA* New York Heart Association

**Table 2:** Quantification of time on hemodialysis, ultrafiltrate volume, and adverse effects related to hemodialysis of the 30 participants, in Hospital Estadual Alberto Rassi, Goiânia, GO, Brazil, from August to December 2022

Parameter	Average	SD
Total session time (h)	3.97	0.13
Time on hemodialysis (months)	6.37	8.17
Total UF (mL)	2,046.67	1,162.26
UFNET (mL)	1,660.00	1,134.90
	**n**	**%**
**Intradialytic hypotension**		
No	23	76.7
Yes	7	23.3
**Catheter bleeding during the session**		
No	29	96.7
Yes	1	3.3
**Symptomatic hypoglycemia during the session**		
No	27	90.0
Yes	3	10.0
**Chest pain during the session**		
No	30	100.0
Yes	0	0
**Nausea and vomiting during the session**		
No	26	86.7
Yes	4	13.3
**Headache during the session**		
No	27	90.0
Yes	3	10.0
**Muscle cramps during the session**		
No	29	96.7
Yes	1	3.3

*SD* standard deviation, *UF* ultrafiltrate, *UFNET* net ultrafiltrate, *n* absolute frequency, *%* relative frequency

**Table 3:** Ultrasound findings, volemic status of each organ, and total sum of the scores of the 30 participants, before and after the hemodialysis session, in Hospital Estadual Alberto Rassi, Goiânia, GO, Brazil, from August to December 2022

Parameter	Evaluation		p*
	Before *n* (%)	After *n* (%)	
**Echocardiography**			
Score + 1	0 (0.0)	4 (13.3)	0.09
Score + 2	23 (76.7)	22 (73.3)	
Score + 3	7 (23.3)	4 (13.3)	
**Number of pulmonary B-lines**			
Score + 1	12 (40.0)	23 (76.7)‡	**0.01**
Score + 2	14 (46.7)	7 (23.3)	
Score + 3	4 (13.3)‡	0 (0.0)	
**IVCCI**			
Score + 1	8 (26.7)	25 (83.3)‡	**< 0.01**
Score + 2	15 (50.0)‡	5 (16.7)	
Score + 3	7 (23.3)‡	0 (0.0)	
**IJVCI**			
Score + 1	15 (50.0)	25 (83.3)‡	**0.02**
Score + 2	9 (30.0)	4 (13.3)	
Score + 3	6 (20.0)‡	1 (3.3)	
**Total score**			
Hypovolemic	9 (30.0)	27 (90.0)‡	**< 0.01**
Normovolemic	16 (53.3)‡	3 (10.0)	
Hypervolemic	5 (16.7)‡	0 (0.0)	

* McNemar’s test, *n* absolute frequency, *%* relative frequency, *IVCCI* inferior vena cava collapsibility index, *IJVCI* internal jugular vein collapsibility index, ‡ posthoc

The comparison of the number of B-lines, IVCCI, IJVCI, and total score of the SAFE-A protocol before and after the hemodialysis session showed statistical significance (Table 3). Also, we observed a change in the volemic profile for all these parameters, since they migrated to more hypovolemic profiles. Although Δechocardiography also showed a change to less hypokinetic profiles, it did not have statistical significance.

**Fig. 1:**
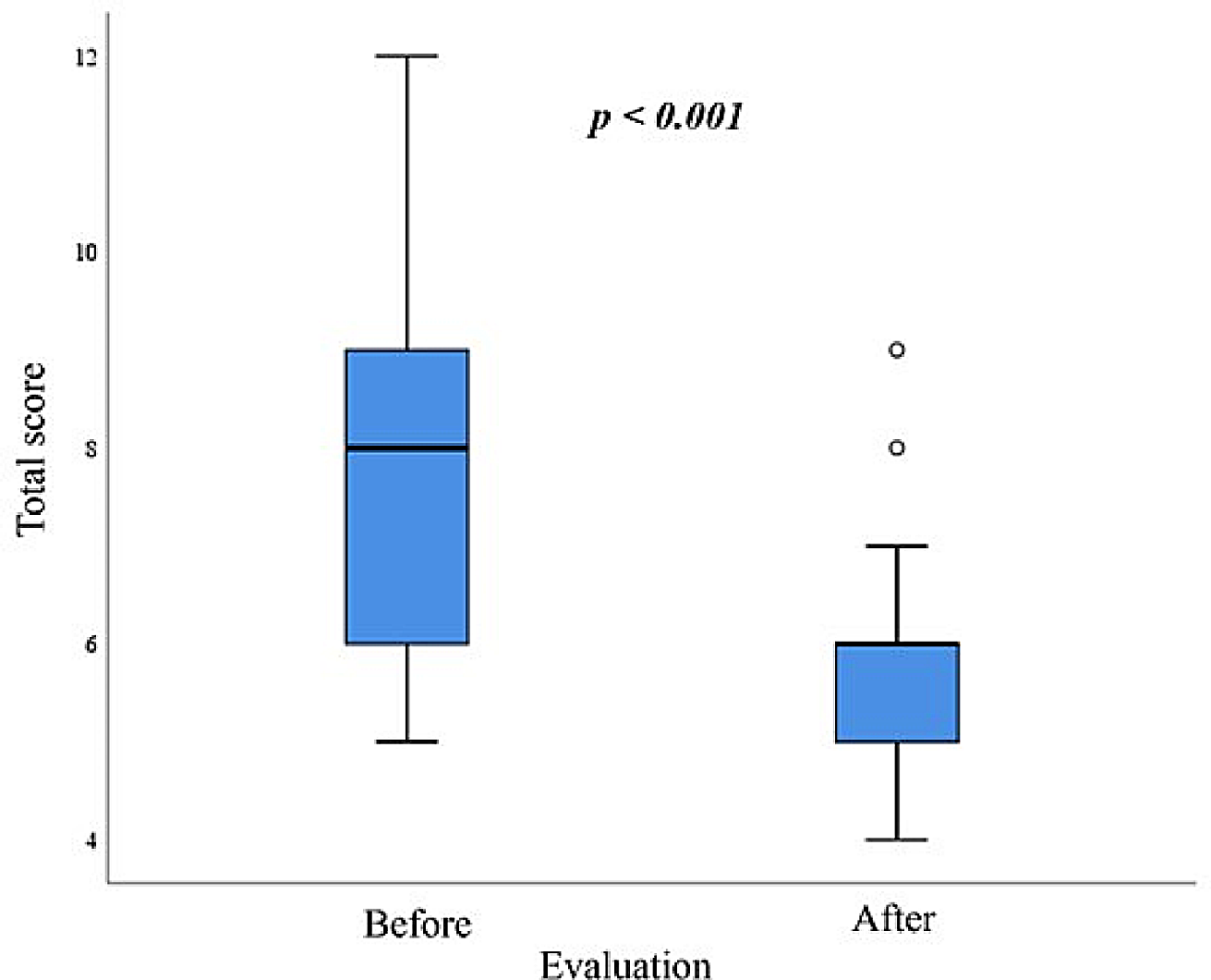
Boxplot graph showing central tendency, symmetry, and dispersion of the total score of the 30 participants, before and after the hemodialysis session, in Alberto Rassi State Hospital, Goiânia, GO, Brazil, from August to December 2022; Paired t-test

A central tendency, symmetry, and dispersion can be observed in Fig. 1. The pre-measurements are less precise and less symmetrical than the post-measurements, demonstrating the power of the hemodialysis session to homogenize the sample. About 75% of the pre-sample was between scores 6 to 9. On average, before the hemodialysis session, the patients were normovolemic (7.63), whereas after it, they were hypovolemic (5.6) (Table 4).

**Table 4:** Comparison of the total sum of the SAFE-A protocol scores of the 30 participants, before and after the hemodialysis session, in Hospital Estadual Alberto Rassi, Goiânia, GO, Brazil, from August to December 2022

Parameter	Evaluation		p*
	Before	After	
Average	7.63	5.60	< 0.01
Standard deviation	1.79	1.10	
Median	8.00	6.00	
Minimum	5.00	4.00	
Maximum	12.00	9.00	

*Paired t-test

Table 5 shows that a negative 1-point ΔSAFE-A had a statistically significant correlation with the withdrawal of 426.73 mL UFNET. Additionally, ΔIJV was statistically significant in isolation with UFNET, but with a higher standard error and lower r² compared to ΔSAFE-A.

**Table 5:** Result of multiple linear regression analysis (backward method) between the net ultrafiltrate and the parameters of the SAFE-A protocol of 30 participants, before and after the hemodialysis session, in Alberto Rassi State Hospital, Goiânia, GO, Brazil, from August to December 2022

Model	Parameter	*r* ^2^	B	Standard error	t	*p*
1	ΔTS	0.37	-426.73	99.98	-4.35	**< 0.01**
2	Δnumber of B-lines	0.41	-327.70	310.99	-1.05	0.30
	Δecocardiography		-120.84	411.38	-0.29	0.78
	ΔIVC		-460.27	292.25	-1.57	0.13
	ΔIJV		-522.38	310.54	-1.68	0.10
3	Δnumber of B-lines	0.37	-331.41	305.22	-1.09	0.29
	ΔIVC		-453.50	286.17	-1.58	0.14
	ΔIJV		-537.84	300.62	-1.79	0,09
4	Δnumber of B-lines	0.35	-350.71	313.40	-1.12	0.27
	ΔIJV		-827.30	245.36	-3.37	**< 0.01**
5	ΔIJV	0.32	-883.87	241.17	-3.66	**< 0.01**

*r*^*2*^ power of correlation; *B* amount of gain in each variation of one point; *t* weight of each variable in significance; *ΔTS* variation of the total score, *Δnumber of B-lines* variation of the number of B-lines, *Δecocardiography* variation of the ecocardiography, *ΔIVC* variation of the inferior vena cava, *Δ*IJV variation of the internal jugular vein

**Fig. 2:**
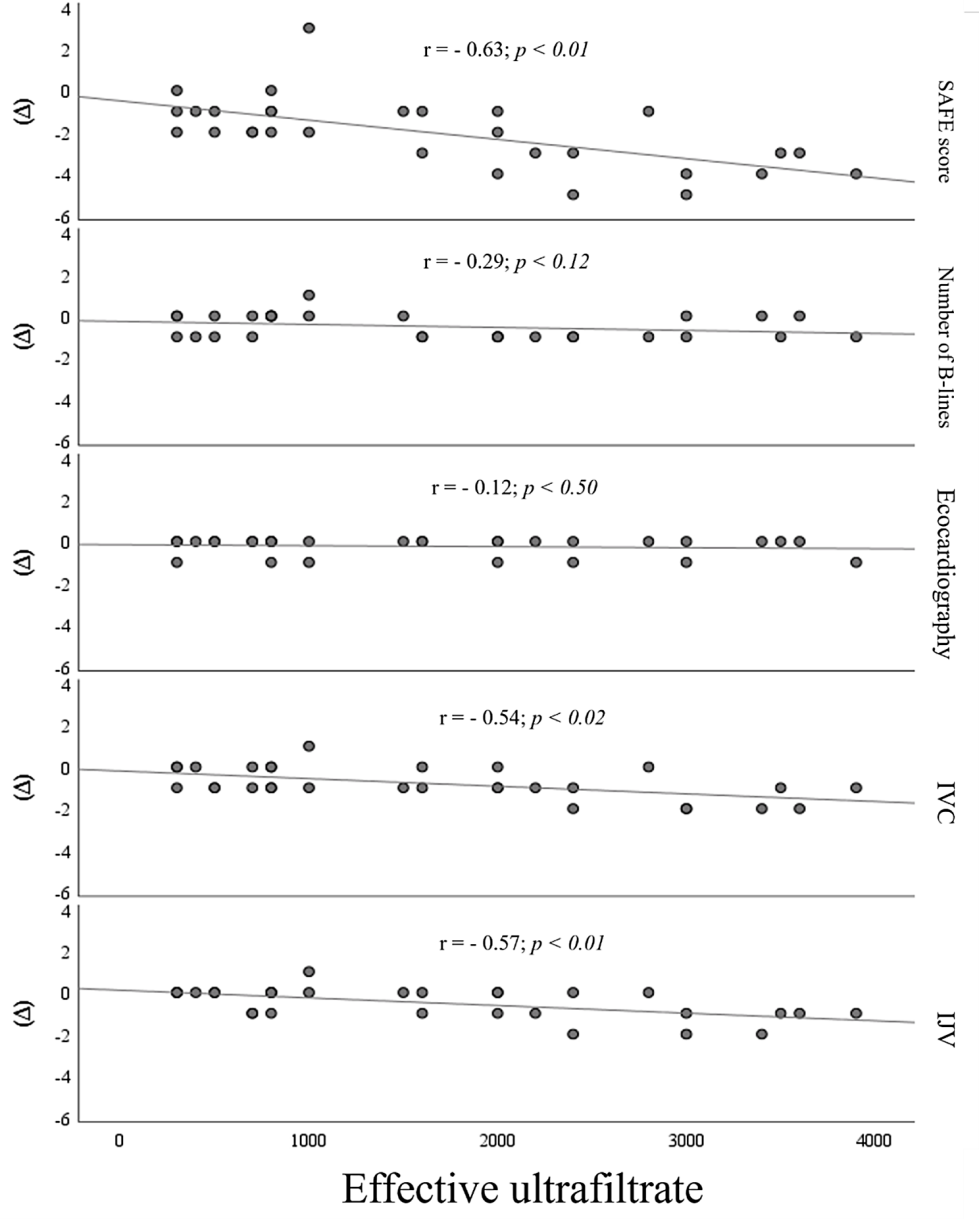
Scatter plot showing the result of Pearson’s correlation between net ultrafiltrate and the variations of the total score, number of B-lines, echocardiography, inferior vena cava, and internal jugular vein of the 30 participants, before and after the hemodialysis session, in Alberto Rassi State Hospital, Goiânia, GO, Brazil, from August to December 2022

**Fig. 3:**
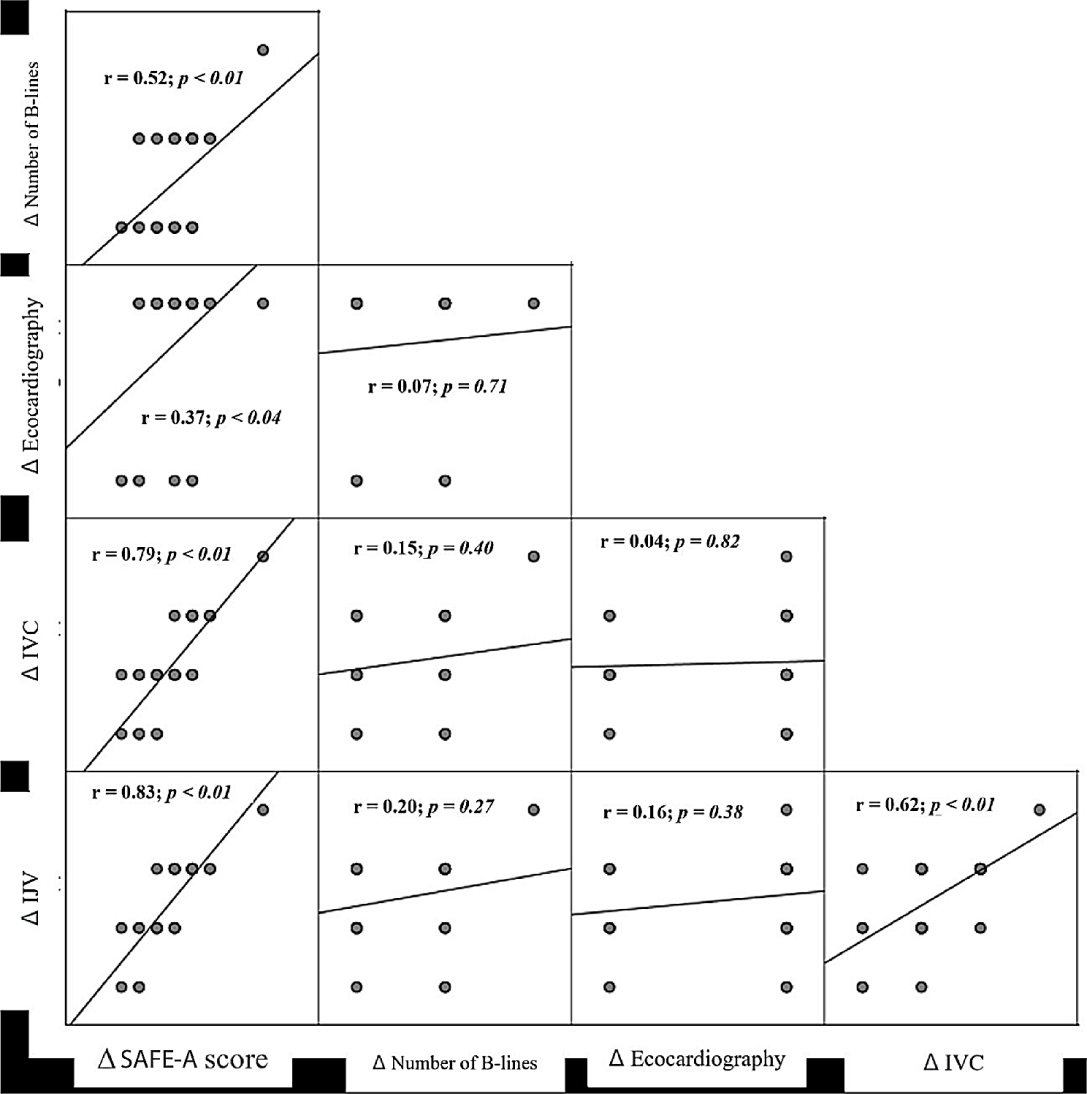
Spearman’s correlation matrix between the variations of the total score, number of B-lines, echocardiography, inferior vena cava, and internal jugular vein of the 30 participants, before and after the hemodialysis session, in Alberto Rassi State Hospital, Goiânia, GO, Brazil, from August to December 2022

Applying Pearson’s correlation between UFNET and the variations of the sonographic findings, a correlation was observed with ΔSAFE-A, ΔIVC, and ΔIJV (Fig. 2). In addition, a Spearman’s correlation matrix was created between the variables themselves, demonstrating a significant correlation between ΔSAFE-A and the other variables, ΔIJV, ΔIVC, Δnumber of B-lines, and Δechocardiography. Furthermore, a positive correlation was found between ΔIJV and ΔIVC (Fig. 3).

Adverse events were observed on 15 participants. Nausea and vomiting on 13.3% (4/30) and IDH on 23% (7/30) during and after hemodialysis.

## Discussion

Pulmonary crackles, alone or combined with peripheral edema, can just reflect interstitial pulmonary edema very poorly in patients presenting with ESDR (Torino et al., 2016) [18]. In contrast, pulmonary ultrasonography can detect asymptomatic pulmonary congestion in hemodialysis patients with greater accuracy than pulmonary auscultation together with peripheral edema or not. Moreover, the number of B-lines has been considered a strong independent predictor of death and cardiac events in this population (Zoccali et al., 2013) [19]. Over 12 months, lung ultrasound-guided dry weight reduction is associated with reversal of cardiac remodeling, regression of myocardial hypertrophy, and amelioration of left ventricle diastolic filling properties (Loutradis et al., 2022) [20]. Furthermore, a lung ultrasound-guided dry-weight reduction performed in outpatients has effectively and safely decreased blood pressure levels in the long term, also generating a lower number of IDH episodes (Loutradis et al., 2021) [21].

Therefore health professionals have tried to find more accurate methods with higher clinical significance for the measurement of the volemic status of hemodialysis patients. Our study meets this trend, demonstrating that, in fact, a significant change occurs in the score of SAFE-A protocol before and after hemodialysis. In addition, we found a correlation between ΔSAFE-A and UFNET. Consequently, the bedside use of SAFE-A protocol can show the volemic profile of these patients and also assist the physician in a more accurate and UFNET withdrawal, although not determining the exact amount of UFNET that should be achieved.

Among the sonographic findings, ΔSAFE-A, ΔIJV, and ΔIVC significantly correlated with UFNET and between each other, but Δechocardiography did not show significant correlation with UFNET, considering that echocardiography does not assess intravascular or extravascular volume and that in this study there was no significant correlation between the net ultrafiltrate variation and the change in echocardiography ultrasound score from the protocol. This lack of association might have occurred because the variation between hypokinesis and hyperkinesis of the cardiac chamber could be lower than that of the other findings, due to the 100% prevalence of ESRD patients and the absence of patients with AKI in our sample. Thus, we assume that, in more chronic patients, the mechanisms of cardiac adaptation would have already been effective, thus reducing this variation, not correlating with fluid withdrawal.

The purpose of diagnostic methods is to reduce adverse events of hemodialysis therapy and, consequently, extra sessions. The limitation of this study lies precisely in the impossibility of tracing a relationship between adverse events in the session, volemic profiles, and UFNET. In addition, we conducted the study with inpatients and these findings cannot be extrapolated to other contexts of medical care.

The strengths of this study were: (1) the reproducibility and acceptability of the method, bearing in mind that we followed norms and techniques to obtain ultrasound windows and measurements; (2) the specific sample of patients on hemodialysis did not present with AKI, making the application of the method more targeted to chronic patients, and therefore more reliable for this population.

This study has several limitations. As it was done in a specific population, the generalization of its findings to any population should not be made. Furthermore, its applicability may be limited depending on the availability of an ultrasound device. Moreover, it would be premature to assert that the implementation of the SAFE-A protocol is inherently safe and beneficial for evaluating dry-weight reduction in end-stage renal disease patients undergoing hemodialysis. Additional research is required to validate its particular utility.

## Conclusion

There was a strong correlation between the score of the SAFE-A protocol and the net ultrafiltrate. Therefore, this study concludes that the application of the SAFE-A protocol in dialysis patients demonstrates a correlation between the suggested score and volume status, consistent with findings from the original study conducted in a distinct population.

## Data Availability

The datasets generated and/or analyzed during the current study are available from Matheus Rabahi, with some restrictions on the data used under license for the current study, which are therefore not publicly available. However, data may be made available by the authors upon reasonable request and with permission from Matheus Rabahi.

## Abbreviations

AKI: Acute kidney injury
B: Amount of gain in each variation of one point
Δecocardiography: Variation of the ecocardiography
ΔIVC: Variation of the inferior vena cava
ΔIJV: Variation of the internal jugular vein
Δnumber of B-lines: Variation of the number of B-lines
ΔSAFE-A: Variation of the score of the SAFE-A protocol
ESRD: End-stage renal disease
IDH: Intradialytic hypotension
IJV: Internal jugular vein
IJVCI: Internal jugular vein collapsibility index
IVC: Inferior vena cava
IVCCI: Inferior vena cava collapsibility index
n: Absolute frequency
NYHA: New York Heart Association
%: Relative frequency
r^2^: Power of correlation
SD: Standard deviation
t: Weight of each variable in significance
UF: Ultrafiltrate
UFNET: Net Ultrafiltrate

## References

[1] Kooman J, Basci A, Pizzarelli F et al (2007) EBPG guideline on haemodynamic instability. Nephrol Dial Transpl 22 Suppl 2:ii22–ii44. 10.1093/ndt/gfm019 10.1093/ndt/gfm019 17507425

[2] Perera P, Mailhot T, Riley D, Mandavia D (2010) The RUSH exam: Rapid Ultrasound in SHock in the evaluation of the critically lll. Emerg Med Clin North Am 28(1):29–56. 10.1016/j.emc.2009.09.010 19945597 10.1016/j.emc.2009.09.010

[3] Kanji HD, McCallum J, Sirounis D, MacRedmond R, Moss R, Boyd JH (2014) Limited echocardiography-guided therapy in subacute shock is associated with change in management and improved outcomes. J Crit Care 29(5):700–705. 10.1016/j.jcrc.2014.04.008 24857642 10.1016/j.jcrc.2014.04.008

[4] Ronco C, Kaushik M, Valle R, Aspromonte N, Peacock IVWF (2012) Diagnosis and management of fluid overload in heart failure and cardio-renal syndrome: the 5B approach. Semin Nephrol 32(1):129–141. 10.1016/j.semnephrol.2011.11.016 22365171 10.1016/j.semnephrol.2011.11.016

[5] Bentzer P, Griesdale DE, Boyd J, MacLean K, Sirounis D, Ayas NT (2016) Will this hemodynamically unstable patient respond to a bolus of intravenous fluids? J Am Med Assoc 316(12):1298–1309. 10.1001/jama.2016.12310 10.1001/jama.2016.12310 27673307

[6] Kaptein MJ, Kaptein EM (2017) Focused real-time ultrasonography for nephrologists. Int J Nephrol 2017:3756857. 10.1155/2017/3756857 28261499 10.1155/2017/3756857 PMC5312502

[7] Brennan JM, Ronan A, Goonewardena S et al (2006) Handcarried ultrasound measurement of the inferior vena cava for assessment of intravascular volume status in the outpatient hemodialysis clinic. Clin J Am Soc Nephrol 1(4):749–753. 10.2215/CJN.00310106 17699282 10.2215/CJN.00310106

[8] Noble VE, Murray AF, Capp R, Sylvia-Reardon MH, Steele DJR, Liteplo A (2009) Ultrasound assessment for extravascular lung water in patients undergoing hemodialysis: Time course for resolution. Chest 135(6):1433–1439. 10.1378/chest.08-1811 19188552 10.1378/chest.08-1811

[9] Mallamaci F, Benedetto FA, Tripepi R et al (2010) Detection of pulmonary congestion by chest ultrasound in dialysis patients. JACC Cardiovasc Imaging 3(6):586–594. 10.1016/j.jcmg.2010.02.005 20541714 10.1016/j.jcmg.2010.02.005

[10] Vitturi N, Dugo M, Soattin M et al (2014) Lung ultrasound during hemodialysis: the role in the assessment of volume status. Int Urol Nephrol 46(1):169–174. 10.1007/s11255-013-0500-5 23884727 10.1007/s11255-013-0500-5

[11] Killu K, Coba V, Blyden D et al (2020) Sonographic Assessment of Intravascular Fluid Estimate (SAFE) score by using bedside ultrasound in the intensive care unit. Crit Care Res Pract 2020:9719751. 10.1155/2020/9719751 32185080 10.1155/2020/9719751 PMC7060409

[12] Killu K, Coba V, Huang Y, Andrezejewski T, Dulchavsky S (2010) Internal jugular vein collapsibility index associated with hypovolemia in the intensive care unit patients. Crit Ultrasound J 2:13–17. 10.1007/s13089-010-0034-3 10.1007/s13089-010-0034-3

[13] Kent A, Patil P, Davila V et al (2015) Sonographic evaluation of intravascular volume status: can internal jugular or femoral vein collapsibility be used in the absence of IVC visualization? Ann Thorac Med 10(1):44–49. 10.4103/1817-1737.146872 25593607 10.4103/1817-1737.146872 PMC4286845

[14] Ilyas A, Ishtiaq W, Assad S et al (2017) Correlation of IVC diameter and collapsibility index with central venous pressure in the assessment of intravascular volume in critically ill patients. Cureus 9(2):e1025. 10.7759/cureus.1025 28348943 10.7759/cureus.1025 PMC5346017

[15] Parikh R, Spring M, Weinberg J, Reardon CC, Farber HW (2019) Use of ultrasound-measured internal jugular vein collapsibility index to determine static intracardiac pressures in patients with presumed pulmonary hypertension. Ann Intensive Care 9(1):124. 10.1186/s13613-019-0595-7 31659483 10.1186/s13613-019-0595-7 PMC6816682

[16] Chambers JM, Cleveland WS, Kleiner B, Tukey P (1983) Graphical methods for data analysis. Wadsworth, Belmont

[17] MacDonald PL, Gardner RC (2000) Type I error rate comparisons of post hoc procedures for I j chi-square tables. Educ Psychol Meas 60(5):735–754. 10.1177/00131640021970871 10.1177/00131640021970871

[18] Torino C, Gargani L, Sicari R et al (2016) The agreement between auscultation and lung ultrasound in hemodialysis patients: the LUST study. Clin J Am Soc Nephrol 11(11):2005–2011. 10.2215/CJN.03890416 27660305 10.2215/CJN.03890416 PMC5108194

[19] Zoccali C, Torino C, Tripepi R et al (2013) Pulmonary congestion predicts cardiac events and mortality in ESRD. J Am Soc Nephrol 24(4):639–646. 10.1681/ASN.2012100990 23449536 10.1681/ASN.2012100990 PMC3609141

[20] Loutradis C, Papadopoulos CE, Sachpekidis V et al (2022) Lung ultrasound-guided dry-weight reduction and echocardiographic changes in clinically euvolemic hypertensive hemodialysis patients: 12-month results of a randomized controlled trial. Hellenic J Cardiol 64:1–6. 10.1016/j.hjc.2021.11.002 34856379 10.1016/j.hjc.2021.11.002

[21] Loutradis C, Sarafidis PA, Ekart R et al (2021) Ambulatory blood pressure changes with lung ultrasound-guided dry-weight reduction in hypertensive hemodialysis patients: 12-month results of a randomized controlled trial. J Hypertens 39(7):1444–1452. 10.1097/HJH.0000000000002818 34074973 10.1097/HJH.0000000000002818

